# Diagnostic performance of a coronary CT angiography-based deep learning model for the prediction of vessel-specific ischemia

**DOI:** 10.1007/s00330-025-12048-4

**Published:** 2025-10-11

**Authors:** Benjamin Peters, Rolf Symons, Sanad Oulkadi, Annelies Van Breda, Yoann Bataille, Peter Kayaert, Willem Dewilde, Kenneth De Wilder, Wouter M. A. Franssen, Alain Nchimi, Olivier Ghekiere

**Affiliations:** 1 UHasselt, School of Medicine and Life Sciences, Agoralaan, 3590 Diepenbeek, Belgium; 2 Department of Radiology, Jessa Ziekenhuis, Stadsomvaart 11, 3500 Hasselt, Belgium; 3 Department of Radiology, Imelda Hospital, Bonheiden, Belgium; 4 Department of Radiology, University Hospital Antwerpen, Edegem, Belgium; 5 Department of Radiology, University Hospital Leuven, Leuven, Belgium; 6 Department of Cardiology, Jessa Ziekenhuis, Hasselt, Belgium; 7 Department of Cardiology, Imelda Hospital, Bonheiden, Belgium; 8 SMRC Sports Medical Research Center, BIOMED Biomedical Research Institute, School of Medicine and Life Sciences, Hasselt University, Diepenbeek, Belgium; 9 GIGA Cardiovascular Sciences, Liège University (ULg), Domaine Universitaire du Sart Tilman, rue de l’Hôpital, Liège, Belgium

**Keywords:** Artificial intelligence, Deep learning, Coronary artery disease, Coronary computed tomography angiography, CT-derived fractional flow reserve

## Abstract

**Objectives:**

Fractional flow reserve (FFR) and instantaneous wave-Free Ratio (iFR) pressure measurements during invasive coronary angiography (ICA) are the gold standard for assessing vessel-specific ischemia. Artificial intelligence has emerged to compute FFR based on coronary computed tomography angiography (CCTA) images (CT-FFR_AI_). We assessed a CT-FFR_AI_ deep learning model for the prediction of vessel-specific ischemia compared to invasive FFR/iFR measurements.

**Materials and methods:**

We retrospectively selected 322 vessels from 275 patients at two centers who underwent CCTA and invasive FFR and/or iFR measurements during ICA within three months. A junior and senior radiologist at each center supervised vessel centerline-building to generate curvilinear reformats that were processed for CT-FFR_AI_ binary outcomes (≤ 0.80 or > 0.80) prediction. Reliability for CT-FFR_AI_ outcomes based on radiologists’ supervision was assessed with Cohen’s *κ*. Diagnostic values of CT-FFR_AI_ were calculated using invasive FFR ≤ 0.80 (*n* = 224) and invasive iFR ≤ 0.89 (*n* = 238) as the gold standard. A multinomial logistic regression model, including all false-positive and false-negative cases, assessed the impact of patient- and CCTA-related factors on diagnostic values of CT-FFR_AI_.

**Results:**

Concordance for CT-FFR_AI_ binary outcomes was substantial (*κ* = 0.725,* p* < 0.001). Sensitivity, specificity, positive predictive value, negative predictive value, and diagnostic accuracy of CT-FFR_AI_ in predicting vessel-specific ischemia on a per-vessel analysis, based on senior radiologists’ evaluations, were 85% (58/68) and 91% (78/86), 82% (128/156) and 78% (119/152), 67% (58/86) and 70% (78/111), 93% (128/138) and 94% (119/127), and 83% (186/224) and 83% (197/238), respectively. Coronary calcifications significantly reduced the diagnostic accuracy of CT-FFR_AI_ (*p* < 0.001; OR, 1.002; 95% CI 1.001–1.003).

**Conclusion:**

CT-FFR_AI_ demonstrates high diagnostic performance in predicting vessel-specific coronary ischemia compared to invasive FFR and iFR. Coronary calcifications negatively affect specificity, suggesting that further improvements in spatial resolution could enhance accuracy.

**Key Points:**

***Question*** *How accurately can a new deep learning model (CT-FFR*_*AI*_) *assess vessel-specific ischemia from CCTA non-invasively compared to two validated pressure measurements during invasive coronary angiography?*

***Findings*** *CT-FFR*_*AI*_ *achieved high diagnostic accuracy in predicting vessel-specific ischemia, with high sensitivity and negative predictive value, independent of scanner type and radiologists’ experience*.

***Clinical relevance*** *CT-FFR*_*AI*_ *provides a non-invasive alternative to Fractional Flow Reserve and instantaneous wave-Free Ratio measurements during invasive coronary angiography for detecting vessel-specific ischemia, potentially reducing the need for invasive procedures, lowering healthcare costs, and improving patient safety*.

**Graphical Abstract:**

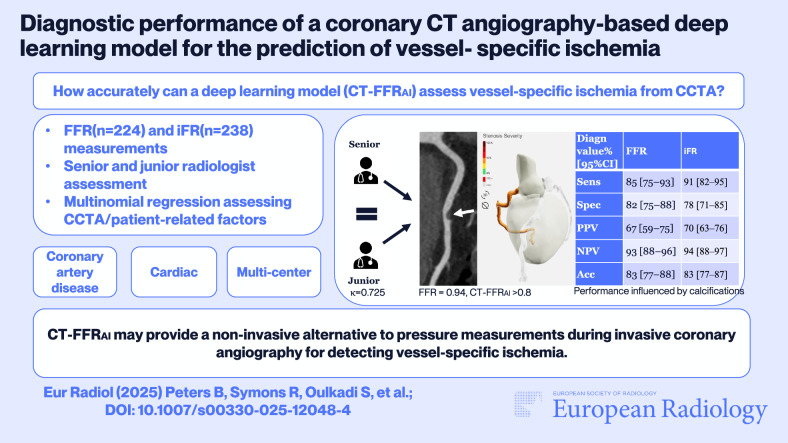

## Introduction

Fractional flow reserve (FFR) during invasive coronary angiography (ICA) is a guideline-endorsed invasive physiological index to assess vessel-specific coronary ischemia. It is defined as the ratio of the mean distal coronary pressure to the mean aortic pressure, measured under maximal hyperemia induced by adenosine, with a threshold of ≤ 0.80 indicating a hemodynamically significant stenosis [1, 2]. Because of the importance of this assessment to guide treatment strategy, other invasive non-hyperemic physiological indices, such as resting instantaneous wave-Free Ratio (iFR), which is calculated by measuring the resting pressure gradient across a coronary lesion during diastole, has been shown to be a reliable alternative at a threshold ≤ 0.89 compared to invasive FFR ≤ 0.80 in recent randomized clinical trials [3, 4].

More recently, emphasis has been placed on the development of non-invasive methods. FFR estimated from computational fluid dynamics (CFD) simulations using coronary computed tomography angiography (CCTA) (CT-FFR) [5] has become an established alternative to invasive FFR and can be considered for the diagnosis and management of patients with intermediate risk and stable chest pain according to recent guidelines [1, 2]. At the same time, artificial intelligence (AI) has been swiftly advancing within the medical field, particularly in diagnostic imaging [6]. Different full- or semi-automated machine learning or deep-learning models (DLMs) were reported to predict invasive FFR from CCTA datasets (CT-FFR_AI_) with an accuracy ranging from 66% to 93%, usually on small single-center cohorts [7]. A new CT-FFR_AI_ model (CorEx, SPIMED-AI) showed a similar diagnostic value to a CFD-based CT-FFR in predicting invasive FFR ≤ 0.80 on a small cohort of patients with coronary stenoses of intermediate severity (40–70% diameter reduction) [8]. In contrast to CFD, a physical model solving the Navier-Stokes equations, this CT-FFR_AI_ model was trained on segmented curved multiplanar reconstruction (cMPR) images integrating a Coronary Artery Disease-Reporting and Data System (CAD-RADS) classification and a binary prediction model (CT-FFR_AI_ ≤ 0.80 or > 0.80) with invasive FFR as ground truth [8, 9]. The purpose of this retrospective multicenter study was to assess the diagnostic performance and reliability of CorEx, Spimed-AI, as CT-FFR_AI_ for the assessment of vessel-specific ischemia as defined by two validated invasive pressure measurements during ICA, in a larger population.

## Materials and methods

### Study population

**Fig. 1:**
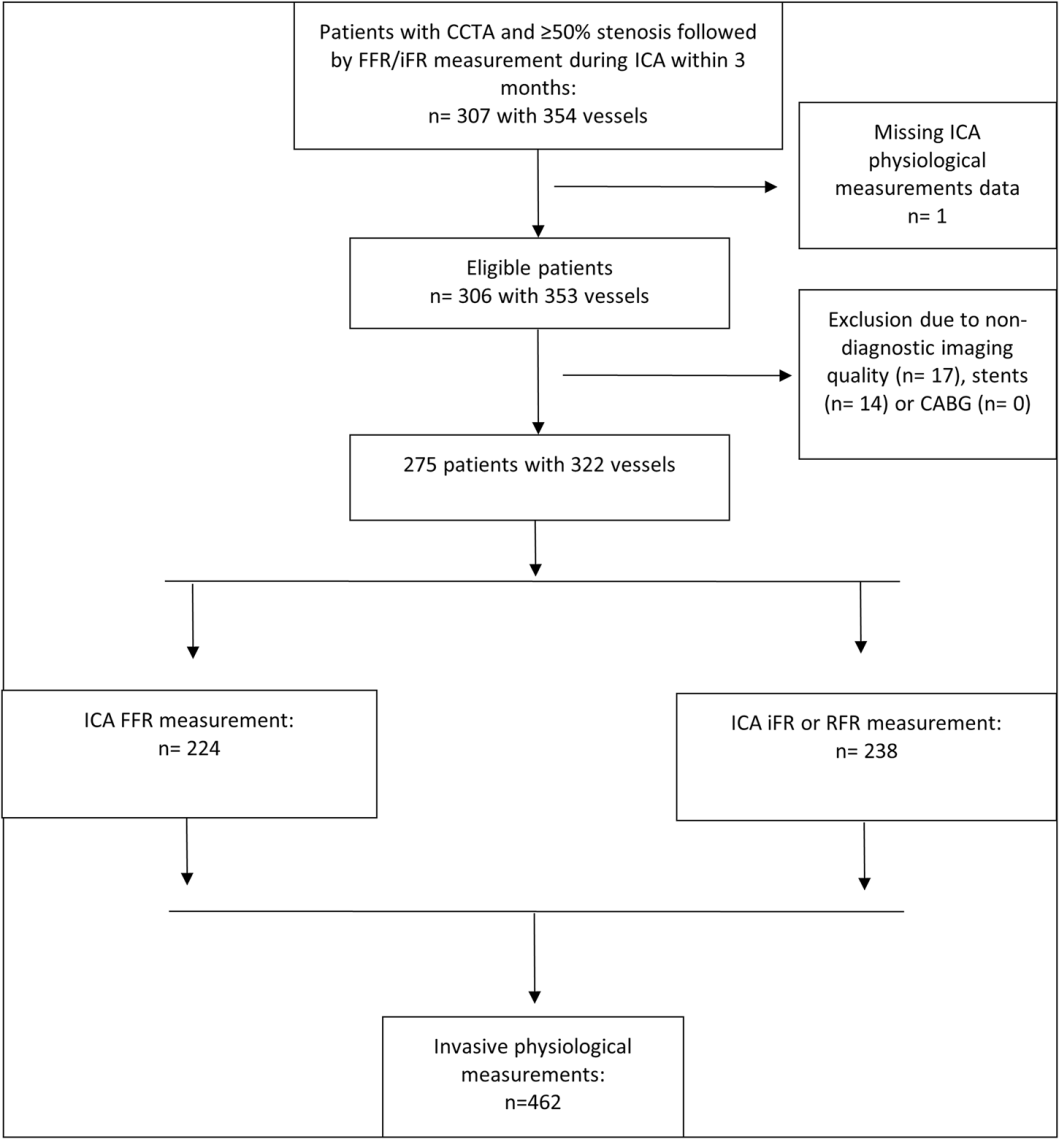
STARD diagram

Our study protocol was approved by the institutional Ethical Review Boards of the participating centers (Center 1: 2022/130, 2022; Center 2: 13/06/2023) and waived the need for informed patient consent in compliance with the law on retrospective analyses of de-identified health data. We retrospectively selected all consecutive patients who underwent CCTA and had a coronary stenosis ≥ 50% on the initial reading, followed by ICA with invasive FFR and/or iFR pressure measurement during ICA within three months, between 2017 and 2022, at two centers. The per-vessel analysis included only coronary arteries with at least one invasive pressure measurement (FFR, iFR, or both) and accounted for patients with multiple vessels showing ≥ 50% stenosis. Vessels without invasive pressure measurement were not included for analysis. CCTA was performed based on the clinical indication of stable chest pain or equivalent symptoms in patients with moderate clinical pre-test likelihood of obstructive coronary artery disease [2]. Exclusion criteria were missing data of ICA assessments (*n* = 1), non-diagnostic imaging quality (*n* = 17), and previous coronary revascularization or bypass grafting on the affected vessel (*n* = 14) (Fig. 1). All eligible patients meeting these criteria were included.

### CT-FFR_AI_ model

The AI-based CCTA FFR prediction model is a DLM that uses nine cMPR images as input and classifies coronary lesions as either CT-FFR_AI_ ≤ 0.80 or > 0.80. The model is trained on CCTA images that have been labeled with FFR values measured during ICA. The model processes nine cMPR images, which were obtained at 40° intervals along the full 360° circumference surrounding the coronary artery centerline, to capture detailed, multi-angle views of the coronary arteries while preserving their natural tortuosity. This 40° interval was determined to provide an optimal balance between accuracy in detecting and quantifying the degree of stenosis in eccentric or calcified lesions, and computational efficiency for AI-based evaluation. At its core, the system employs a convolutional network inspired by the InceptionV3 architecture to extract key image features directly from the data [10]. Instead of relying on a single model, the approach is structured around several intermediate models, each dedicated to a specific function. These models help refine the analysis by detecting calcification, evaluating a CAD-RADS 6-class classification score, which indicates the degree of stenosis, and assessing overall image quality [9, 11]. Their outputs provide additional context that strengthens the final classification, particularly since clinical evidence demonstrates a strong correlation between CAD-RADS and invasive FFR [12]. To optimize performance, these intermediate models are initialized with weights from a pre-trained single-image model, streamlining and accelerating the training process while ensuring consistency across the different tasks. Unlike traditional methods that depend on solving the partial differential equations of CFD to model blood flow and pressure, our model learns the relationship between coronary anatomy and FFR directly from the images. This direct learning method eliminates the need for complex simulations, thereby enabling fast, real-time analysis in a clinical setting. To further enhance transparency, the model uses integrated gradients to highlight the specific regions in the CCTA images that most influence its predictions for CAD-RADS, which clarifies the decision-making process. Unlike other AI models focusing on 3D volumetric analysis or CFD alone, CT-FFR_AI_ integrates anatomical and functional data through advanced deep-learning techniques, enhancing prediction robustness.

### CT acquisition and image processing

**Fig. 2:**
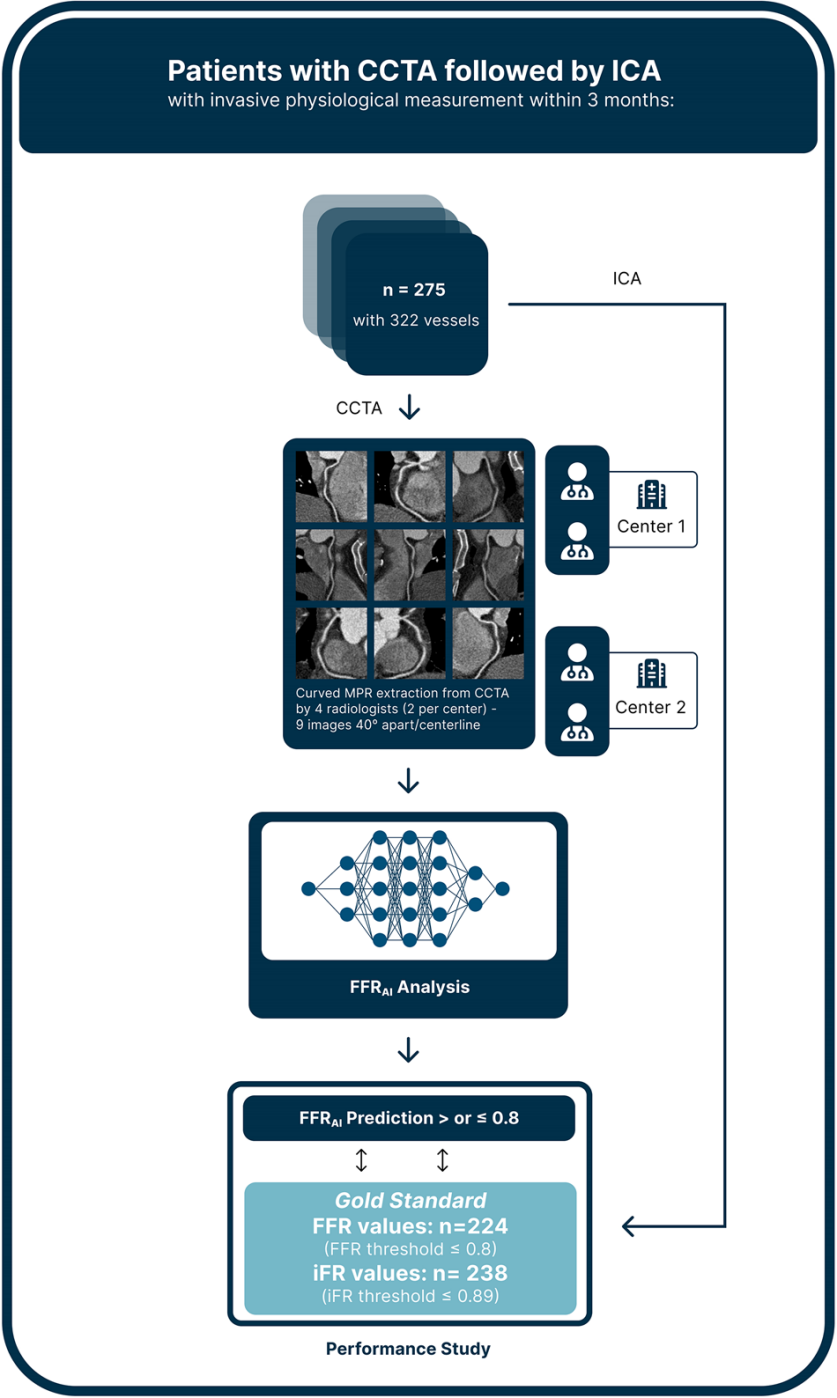
Study workflow. All coronary computed tomography angiography (CCTA) examinations were evaluated by a senior and junior radiologist at each center for centerline-building supervision on all stenotic segments. A total of nine curved multiplanar reconstruction images of each coronary artery were uploaded to CorEx (Spimed-AI), a CCTA-based deep-learning model for invasive Fractional Flow Reserve prediction (CT-FFR_AI_). These images were subsequently analyzed to assess the diagnostic performance of CT-FFR_AI_, with invasive Fractional Flow Reserve ≤ 0.8 and invasive instantaneous wave-Free Ratio ≤ 0.89 as ground truth

Patients underwent CCTA with a 320-detector-row CT scanner (Aquilion One Vision Edition, Canon Medical System Corporation) in Center 1 and a 96-detector-row dual source CT scanner (Somatom Force, Siemens Healthineers) in Center 2. Scanning and contrast administration parameters of both centers are given in Table 1. Non-contrast imaging was performed to calculate the Agatston score at 120 kV and 3-mm slice thickness in Center 1 and Sn100 kV and 3-mm slice thickness with virtual 120 kV reconstructions in Center 2. The Agatston score was computed on non-contrast-enhanced CT images using dedicated software (Syngo via B60, Siemens Healthineers). Calcified lesions were identified as areas with attenuation values ≥ 130 Hounsfield units (HUs) and an area ≥ 1 mm². Calcifications were automatically detected and manually verified by senior radiologists. The Agatston score for each coronary vessel was derived by summing the calcifications of the coronary artery. Subsequent CCTA acquisition was performed with prospective or retrospective electrocardiographic gating, according to the Society of Cardiovascular Computed Tomography guidelines [13]. The best diastolic or systolic phase was selected by the local investigator and sent to a dedicated software (Syngo via B60, Siemens Healthineers) for analysis by a senior (B.P., 10 years’ experience and R.S., 11 years’ experience in Centers 1 and 2, respectively) and a junior (S.O.,3 years in-training and A.VB., 2 years in-training in Centers 1 and 2, respectively) radiologist, blinded to patients’ information, clinical data, and invasive pressure measurement values during ICA. CMPR images along the main coronary arteries were automatically reconstructed using standardized window settings (width: 1000 HU, level 300 HU). The initial centerline placement and windowing parameters were generated by the software but were subject to manual adjustments by either senior or junior radiologists as deemed necessary. Notably, no additional training was provided for these manual modifications, which could potentially influence the results of CT-FFR_AI_. Subsequently, a total of nine cMPR images of each coronary artery were uploaded to a server-based module, CorEx, for CT-FFR_AI_ prediction (Fig. 2). These images were subsequently analyzed to assess the diagnostic performance of CT-FFR_AI_, as per-vessel centerline-building supervised by either senior or junior radiologists. Senior radiologists evaluated the influence of several patient- and CCTA image-related parameters on the diagnostic accuracy of CT-FFR_AI_ in predicting vessel-specific ischemia. A region of interest was placed in the ascending aorta and the right main bronchus, such that it included the maximum vessel or bronchial lumen, carefully avoiding its limits, to measure the mean attenuation and standard deviation (SD). The CCTA signal-to-noise ratio (SNR) was calculated as the ratio between the mean attenuation in the ascending aorta and the SD of the attenuation in the right main bronchus. The minimal cross-sectional diameter of all coronary arteries was obtained at the origin of each vessel in a cross-sectional reformation along the long axis of the vessel through the software’s automatic measuring function, as previously reported [14].

**Table 1:** Technological and acquisition parameters for calcium score and CCTA imaging in both centers

		Center 1	Center 2
Number of X-ray tubes		1	2
Number of detectors		320	96
Gantry rotation speed		275 ms	250 ms
Calcium score	Tube voltage	120 kV	Sn100 kVp
	Slice thickness	3 mm	3 mm
	Reconstruction algorithm	AIDR3D	ADMIRE 2
	Reconstruction kernel	FC12	Sa36f
CCTA	Tube voltage	100–120 kV	80–120 kVp
	Slice thickness	0,5 mm	0,6 mm
	Acquisition	Prospective	Prospective/retrospective
	DLP	140 (91.6–194.8) (mGy.cm)	54.9 (37.5–106.2) (mGy.cm)
	Reconstruction algorithm	AIDR3D	ADMIRE 2
	Reconstruction kernel	FC03	Bv40 or Bv49
	Reconstruction matrix	512 × 512 matrix	512 × 512 matrix
	Pitch	N/A	0.2–3.2
Contrast administration:		Ultravist 370	Xenetix 350
	Injection rate and volumes	^a^100 kV, < 450 mA: 45 mL @ 5 mL/s	^a^80–100 kVp: 67 mL @ 3.7 mL/s
		^a^100 kV, 450–900 mA: 55 mL @ 6 mL/s	^a^110–120 kVp: 76 mL @ 4.5 mL/s
		^a^120 kV: 65 mL @ 7 mL/s	^a^130–140 kVp: 85 mL @ 5.0 mL/s

Data are expressed as median and interquartile range (IQR)

*CCTA* coronary computed tomography angiography, *kV* tube voltage,* kVp* tube peak voltage, *mA* milliampere, *AIDR3D* adaptive iterative dose reduction, Canon Medical System Corporation, *ADMIRE 2* advanced modeled iterative reconstruction, Siemens Healthineers, *FC* filter convolution, *Sa36f* calcium-aware kernel, Siemens Healthineers, *Bv* bone viewing, *DLP* dose length product, *FOV* field of view, *N/A* not applicable

^a^ Injection rate (3.7–7 mL/s) and volumes (45–85 mL) were optimized on a patient basis, according to the tube voltage and current determined by the automatic exposure control (SUREexposure3D, Canon Medical Systems and Care Dose4D, Siemens Healthineers) using the subject’s body surface area

### Invasive coronary angiography assessments

The luminal stenosis of coronary disease on ICA was evaluated by two senior interventional cardiologists (P.K., 13 years’ experience and Y.B., 12 years’ experience in Center 1; W.D., 18 years’ experience and K.D., 8 years’ experience in Center 2) at each center blinded to patients’ information, clinical data and CT-FFR_AI_ assessment, and was classically categorized into 4 degrees on the basis of the cross-sectional stenosis severity: 0–25%, 26–50%, 51–75%, and 76–100% [15]. Discrepancies in categorizing were assessed by consensus. Invasive FFR and iFR measurements during ICA were performed by a local interventional cardiologist, according to recent guidelines [1, 2]. Resting full-cycle ratio measurements were considered similar to invasive iFR at a 0.89 threshold, according to Svanerud J et al [16]. Patients presenting with chronic coronary syndrome or equivalent symptoms and uncertainty regarding the hemodynamic significance of coronary stenosis were considered for invasive physiological assessment using invasive FFR, iFR, or both. While these indices often yield concordant results, discrepancies can occur due to factors such as microvascular obstruction, microvascular dysfunction, or aortic stenosis, which may influence both measurements. The combined use of invasive FFR and iFR facilitates the identification of such cases, particularly in complex or borderline stenoses. In instances where FFR and iFR results were discordant, treatment decisions were based on a comprehensive evaluation of the patient’s clinical history, presenting symptoms, and any available non-invasive test results [3, 4].

### Statistical analysis

Statistical analyses were performed (W.MA.F.) using SPSS v 29.0. The sample size calculation was performed using R version 4.4.1 (The R project for Statistical Computing) and based on a study of Coenen et al, who showed an accuracy of 78%, a sensitivity of 82% and a specificity of 76% [17]. Assuming a statistical power > 0.8, a two-sided alpha of 0.0,5 and a 0.35 proportion of patients with vessel-specific coronary ischemia, a total of at least 309 vessel measurements had to be included in the present study. Continuous data with a normal distribution (based on Shapiro–Wilk tests) are expressed as the means ± SD, whereas data with a non-normal distribution are expressed as median and interquartile range (IQR), and categorical variables are presented as frequency and percentage.

Each senior and junior radiologist evaluated the cases from their own center, and statistical analyses were performed on pooled data. Concordance between junior and senior evaluations of CT-FFR_AI_ binary outcomes (≤ 0.80 or > 0.80) resulting from their respective centerline-building supervision was calculated using Cohen’s *κ* as follows: values ≤ 0 as indicating no reliability, 0.01–0.20 as none to slight, 0.21–0.40 as fair, 0.41–0.60 as moderate, 0.61–0.80 as substantial, and 0.81–1.00 as almost perfect.

The diagnostic values of CT-FFR_AI_ evaluated by both junior and senior radiologists, using invasive FFR or iFR as the standard of reference for vessel-specific ischemia, were expressed as sensitivity, specificity, positive predictive value (PPV), and negative predictive value (NPV), and calculated with 95% confidence intervals (CIs). When both invasive FFR and iFR values were obtained, they were first analyzed independently. A subgroup analysis was performed on vessels with both invasive FFR and iFR measurements (*n* = 140). The diagnostic accuracy of CT-FFR_AI_ between senior and junior radiologists was compared by applying the chi-square test. The final reported diagnostic CT-FFR_AI_ values in the Results and Discussion section will pertain to senior radiologists unless otherwise stated.

Patient- and CCTA image-related parameters associated with false-positive and false-negative CT-FFR_AI_ analyses were evaluated using a multinomial logistic regression model. A backward stepwise logistic regression was performed to identify significant predictors of false-positive and false-negative outcomes while controlling for potential confounders. The model initially included all independent variables. Non-significant variables were systematically removed based on the likelihood ratio test, using a removal criterion of *p* > 0.05. Selection was based on odds ratios (ORs) for patient-related factors (age, gender, body mass index (BMI), heart rate during acquisition, calcium score of the stenotic vessel, and ostial diameter of the stenotic coronary artery) and technical factors (SNR, CT scanner type, and time between CCTA and invasive FFR/iFR). A two-tailed *p*-value of < 0.05 indicated statistical significance.

## Results

### Patient and coronary stenosis characteristics

In total, 275 patients (mean age 66 ± 9; range 37–91 years) met the criteria for inclusion in this retrospective study, including 187 males and 88 females. Thirty-two patients were excluded due to missing ICA pressure measurement data (*n* = 1), poor CCTA imaging quality (*n* = 17), and previous coronary revascularization (*n* = 14) of the affected vessel (Fig. 1). Median BMI was 26.9 (IQR 23.9–29.4) kg/m², and median heart rate during CCTA acquisition was 60 (IQR 55–66) bpm. Demographics and cardiovascular risk factors are given in Table 2. The mean time interval between CCTA and invasive FFR and/or iFR measurements during ICA was 26 ± 20 (range 0–91) days. In total, 462 pressure measurements performed during ICA, including 224 invasive FFR measurements (median FFR value 0.85; IQR 0.79–0.89) and 238 invasive iFR measurements (median iFR value 0.91; IQR 0.86–0.95) evaluations in 322 stenotic vessels, were available for comparison with CT-FFR_AI_. Forty-three percent (140/322) of coronary stenoses were evaluated both with invasive FFR and invasive iFR. Upon consensus reading of ICA, there were 25 stenoses in the 0–25% range, 185 in the 26–50% range, 207 in the 51–75% range, and 37 in the 76–100% range. Data were missing for the grading of 8 coronary stenoses.

**Table 2:** Patient characteristics and cardiovascular risk factors

	Number (%)	Mean ± SD (range)/median [IQR]
Patients (*n*)	275	
Male (*n*)	187 (68)	
Female (*n*)	88 (32)	
Age (years)		66 ± 9 [37–91]
BMI (kg/m²)		26.9 (23.9–29.4)
Systemic hypertension	174 (63)	
Current tobacco smoker	78 (28)	
Diabetes mellitus	44 (16)	
Family history of coronary disease	114 (41)	
Prior coronary treatment	10 (4)	
HR during acquisition (bpm)		60 (55–66)
Agatston coronary calcium score		356.8 (122.7–658.8)

*SD* standard deviation, *IQR* interquartile range, *BMI* body mass index, *HR* heart rate, *bpm* beats per minute

### Diagnostic value of CT-FFR_AI_

The concordance for CT-FFR_AI_ binary outcomes (≤ 0.80 or > 0.80) between senior and junior radiologists’ evaluations was substantial (*κ* = 0,725; *p* < 0.001). The accuracy of CT-FFR_AI_ in predicting invasive FFR ≤ 0.80 and invasive iFR ≤ 0.89 as per senior radiologists’ centerline-building supervision was 83% (186/224) and 83% (197/238), respectively, slightly higher than79% (177/224) and 78% (186/238), respectively, per junior radiologists’ supervision. The difference in diagnostic accuracy of CT-FFR_AI_ between senior and junior radiologists’ supervision was not significant (*p* = 0.327). All diagnostic values are listed in Table 3. However, the diagnostic accuracy of unsupervised CT-FFR_AI_ dropped to 61% (136/224) and 62% (148/238) compared to invasive FFR and iFR, respectively (*p* < 0.001). There was notably lower specificity and PPV due to the increase in false-positive cases. All diagnostic values without radiologists’ supervision are provided in a Supplementary Table A.

**Table 3:** The diagnostic values of CT-FFR_AI_ based on binary outcome analysis compared to invasive FFR and iFR measurements

Senior reader			Junior reader	
Diagnostic value% (95% CI)	FFR (*n* = 224)	iFR (*n* = 238)	FFR (*n* = 224)	iFR (*n* = 238)
Sensitivity	85 (58/68) [75–93]	91 (78/86) [82–95]	79 (54/68) [68–88]	80 (69/86) [70–88]
Specificity	82 (128/156) [75–88]	78 (119/152) [71–85]	79 (123/156) [72–85]	77 (117/152)[69–83]
PPV	67 (58/86) [59–75]	70 (78/111) [63–76]	62 (54/87) [54–69]	66 (69/104) [59–73]
NPV	93 (128/138) [88–96]	94 (119/127) [88–97]	90 (123/137) [85–93]	87 (117/134) [82–91]
Accuracy	83 (186/224) [77–88]	83 (197/238) [77–87]	79 (177/224) [73–84]	78 (186/238) [72–83]

The table shows the diagnostic values of CT-FFR_AI_ evaluated by both junior and senior radiologists and using invasive FFR and/or iFR as the standard of reference for vessel-specific ischemia. When both invasive FFR and iFR values were obtained, they were analyzed independently. Diagnostic values for senior and junior radiologists are given separately

*CT-FFR*_*AI*_ coronary computed tomography angiography-based artificial intelligence deep-learning model for the prediction of invasive Fractional Flow Reserve, *FFR* Fractional Flow Reserve, *iFR* instantaneous wave-Free Ratio, *95% CI* 95% confidence interval, *PPV* positive predictive value, *NPV* negative predictive value

In lesions assessed with both invasive FFR and iFR (*n* = 140), senior radiologist-supervised CT-FFR_AI_ demonstrated similar diagnostic accuracies of 85% (119/140) and 80.7% (113/140), respectively, comparable to those observed in the overall cohort. Discordance between invasive FFR and iFR measurements, defined as FFR ≤ 0.80 and iFR > 0.89 (*n* = 7) or FFR > 0.80 and iFR ≤ 0.89 (*n* = 9), was observed in 11% of cases (16/140). In this discordant subgroup, senior radiologist-supervised CT-FFR_AI_ agreed with FFR in 69% (11/16) and with iFR in 31% (5/16).

### Value of CT-FFR_AI_ in relation to morphological stenosis severity

**Fig. 3:**
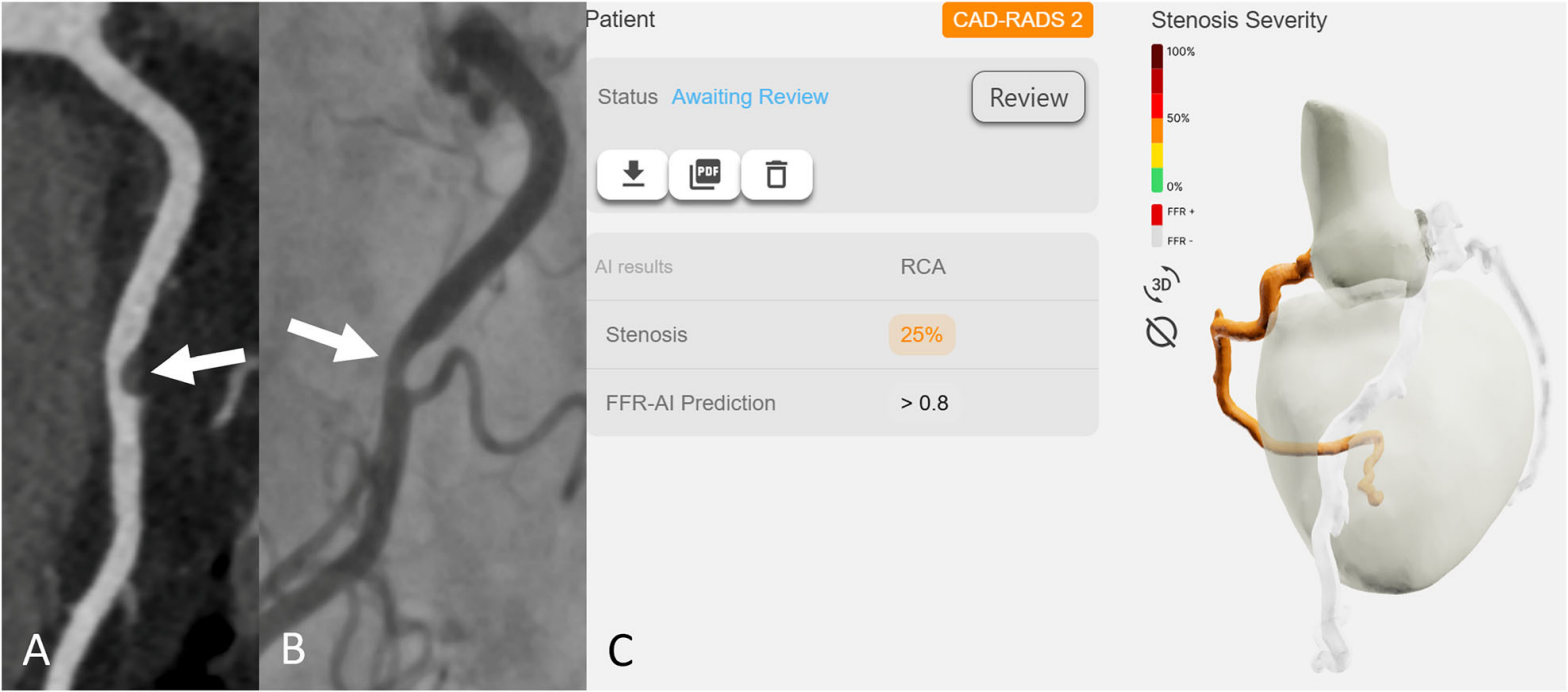
True-negative case. A representative case of a 60-year-old female patient with a recent onset of stable chest pain. Curved multiplanar reconstruction coronary computed tomography angiography **A** shows a soft plaque at the middle portion of the right coronary artery with obstructive stenosis (white arrow). Invasive coronary angiography **B** confirms a 26–50% stenosis (white arrow). The Fractional Flow Reserve measurement was 0.94, which is above the 0.8 threshold. Instantaneous wave-Free Ratio measurement was 0.95, which is above the 0.89 threshold. The CT-FFR_AI_ prediction was > 0.8 (**C**)

**Fig. 4:**
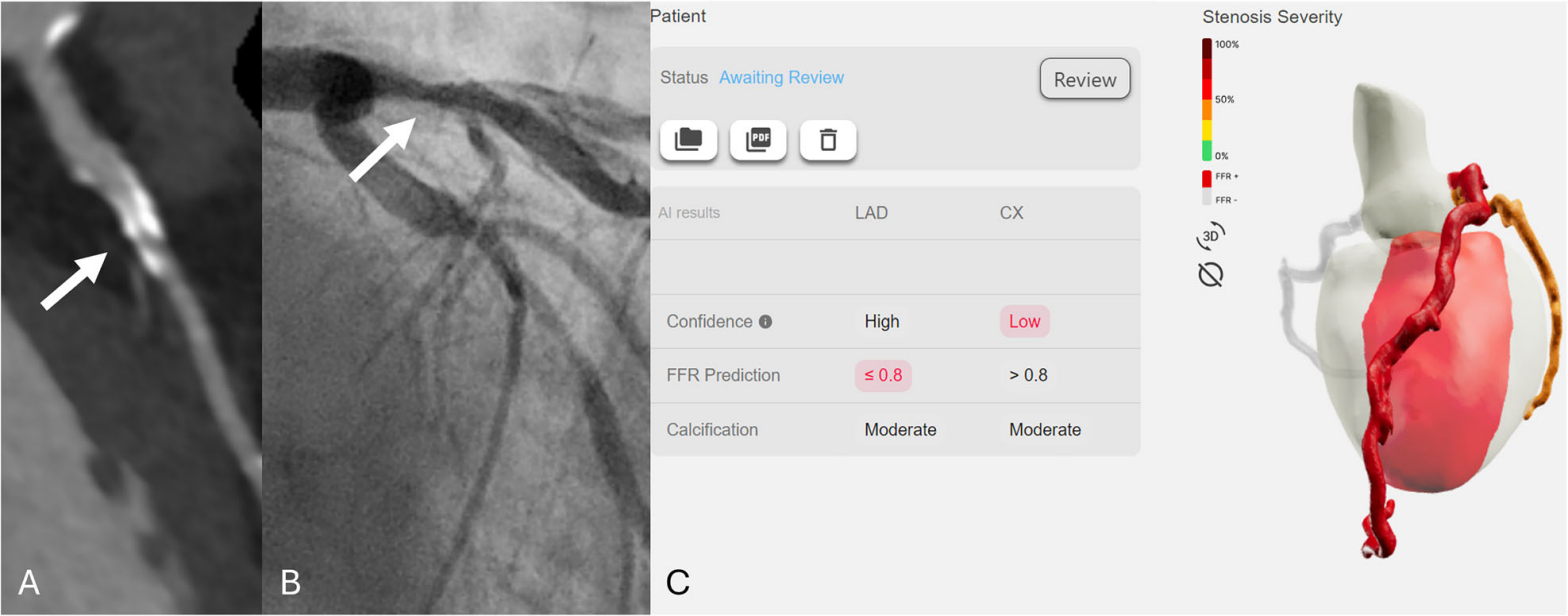
False-positive case. A representative case of an 81-year-old male patient with a recent onset of stable chest pain. Curved multiplanar reconstruction coronary computed tomography angiography **A** shows a heavily calcified plaque with blooming artifacts (white arrow) in the proximal left anterior descending artery with obstructive stenosis. Invasive coronary angiography **B** demonstrates a 26–50% stenosis, and Fractional Flow Reserve was 0.89, in contrast to the report of CT-FFR_AI_ that predicts FFR ≤ 0.8 (**C**). Of note, the patient also had an obstructive stenosis on the left circumflex artery with CT-FFR_AI_ > 0.8 prediction and invasive Fractional Flow Reserve > 0.8 (FFR = 0.91)

The case-by-case contingency table for the accuracy of CT-FFR_AI_ prediction of vessel-specific ischemia, as per senior radiologists’ centerline-building supervision and according to stenosis severity on ICA, is provided in Table 4. Figure 3 illustrates a representative example of a true-negative case. All ≤ 25% stenosis were classified as CT-FFR_AI_ > 0.80, while only three coronary stenoses > 75% on ICA had a false-positive CT-FFR_AI_ analysis, which had a high calcium score of the stenotic vessel (Agatston scores of respectively 357, 317, and 317). The remaining 58 false-positive cases were in the 26–75% ICA stenosis range (Fig. 4). A total of 18 cases had a false-negative CT-FFR_AI_ prediction, all in the 26–75% ICA stenosis range. 16/18 of these false-negatives were also within the gray-zone ranges for invasive FFR 0.75–0.80 (*n* = 9) and invasive iFR 0.86–0.89 (*n* = 7); while one stenotic vessel had an invasive FFR measurement of 0.71 and an iFR measurement of 0.81 during ICA.

**Table 4:** CT-FFR_AI_ case-by-case assignment relative to vessel-specific ischemia based on senior radiologists' supervision and invasive coronary angiography stenosis severity

Stenosis severity on the ICA	Number (%)	TP	TN	FP	FN
0–25%	25 (5)	0	25	0	0
26–50%	185 (40)	19	128	30	8
51–75%	207 (45)	89	80	28	10
76–100%	37 (8)	28	6	3	0
Unknown^a^	8 (2)	0	8	0	0
Total	462	136	247	61	18

*CT-FFR*_*AI*_ coronary computed tomography angiography-based artificial intelligence deep-learning model for the prediction of invasive Fractional Flow Reserve, *TP* true positive, *TN* true negative, *FP* false-positive, *FN* false-negative

^a^ Unknown: missing images of invasive coronary angiography for re-evaluation of coronary artery stenosis severity by two interventional cardiologists

The total number of cases includes invasive FFR and iFR measurement when both were used

### Effect of patient- and CCTA image-related factors

Only the calcium score of the stenotic vessel had a significant detrimental effect on the diagnostic accuracy of CT-FFR_AI_ prediction of vessel-specific ischemia. This was demonstrated using a multinomial logistic regression analysis based on all false-positive and false-negative cases (*p* < 0.001; OR, 1.002; 95% CI 1.001–1.003), and based on all false-positive cases alone (*p* < 0.001; OR, 1.002; 95% CI 1.001–1.003). Scanner type and all other patient-related factors, such as age, gender, BMI, HR during acquisition, and ostial diameter of the stenotic coronary artery, had no significant influence on the diagnostic accuracy of CT-FFR_AI_ (Table 5).

**Table 5:** Multinomial logistic regression model with backward variable selection to identify patient- and CCTA image-related factors influencing the diagnostic performance of CT-FFR_AI_

Parameters	Mean ± SD (range)/median (IQR)	OR	95% CI	*p*-value
Age	67 (60–74) years	0.995	0.979–1.010	0.496
Gender	312 males	0.850	0.480–1.507	0.579
BMI	26.6 (24.28–29.4) kg/m^2^	1.048	0.982–1.118	0.159
Time between CCTA and intracoronary measurement	22 (12–37) days	1.000	0.988–1.012	0.944
CT scanner (147 AQ and 315 SF)		0.588	0.273–1.266	0.175
HR during CT acquisition	59 (54–65) bpm	0.985	0.956–1.015	0.325
Image SNR	21.11 (16.41–27.48)	1.009	0.972–1.048	0.628
Calcium score (Agatston) of the stenotic vessel	169.1 (42.8–348.1)	1.002	1.001–1.003	< 0.001
Minimal diameter	3.6 (3.2–4.1) mm	0.711	0.451–1.118	0.140

*CCTA* coronary computed tomography angiography, *CT-FFR*_*AI*_ coronary computed tomography angiography-based artificial intelligence deep-learning model for the prediction of invasive Fractional Flow Reserve, *SD* standard deviation, *IQR* interquartile range, *SNR* signal to noise ratio, *BMI* body mass index, *HR* heart rate, *AQ* 320-detector-row CT scanner Aquilion One Vision Edition, Canon Medical System Corporation, *SF* 96-detector-row dual source CT scanner Somatom Force, Siemens Healthineers, *bpm* beats per minute

Odd ratios are computed based on all false-positives and false-negatives (*n* = 79)

## Discussion

In this large cohort multicenter study, CT-FFR_AI_ demonstrated an 83% diagnostic accuracy for the prediction of vessel-specific coronary ischemia as defined by two validated methods of pressure measurements during ICA, namely invasive FFR and iFR [3, 4]. The model’s reliability was demonstrated by a similar diagnostic performance using datasets from 2 different CT scanners, encompassing both prospective and retrospective scanning modes, and by substantial observer concordance between supervisors of different levels of experience. The diagnostic accuracy of our CT-FFR_AI_ aligns with that of an established CFD-based CT-FFR (71–92%) [5, 18–21] and with a recent quantitative CCTA-based AI model reporting 72-82% accuracy [22]. Importantly, in intermediate-grade stenoses, a clinical subset where decision-making is most challenging, CFD-based CT-FFR accuracy in estimating vessel-specific ischemia dropped to 69% on a per-vessel basis and to 73% on a per-patient basis [21]. In contrast, our CT-FFR_AI_ maintained a diagnostic accuracy between 77–88% (95% CI), which is consistent with the results from a proof-of-concept study using the same AI model on intermediate-grade stenosis [8]. However, the diagnostic accuracy of CT-FFR_AI_ dropped significantly without radiologist supervision, primarily due to an increased rate of false-positive cases. This highlights the current need for supervised CT-FFR_AI_ analysis. Other AI models reported 66–79% accuracy in predicting ischemia using 3D volumetric heart images, a fully automated 3D DLM, or even different invasive FFR thresholds [19, 23, 24]. All these models had different underlying algorithms to evaluate vessel-specific coronary ischemia with a continuous or binary outcome. Coenen et al combined machine learning with CFD, achieving 78% accuracy per-vessel in a multicenter study of 351 patients [17]. The higher accuracy of our CT-FFR_AI_ compared to other models might be due to the integration of two DLMs, including anatomical and functional information. One was trained to provide stenosis CAD-RADS 6-class classification, and the second to predict the 0.8 FFR threshold, considering the known nonlinear relationship between invasive FFR values and stenosis severity [8, 11]. Using this combined approach could help prioritize high-risk examinations in daily clinical practice and potentially increase efficiency and workload [25–27]. Although CFD-based CT-FFR is becoming more established, its clinical adoption remains limited because of the substantial time required to simulate hemodynamic changes, with reported processing times ranging from 10 min to several hours depending on computational resources [5, 17]. In contrast, CT-FFR_AI_ utilizes a local server-based DLM enabling nearly real-time analysis in clinical settings, although direct timing comparisons were not performed [8, 27].

The high NPV of CT-FFR_AI_ suggests its potential to reduce the need for further ICA measurements, thereby lowering costs and avoiding the risks associated with invasive procedures [28]. The high NPV of CT-FFR_AI_ is further supported by the fact that nearly 90% (16/18) of false-negative cases in our study had either invasive FFR values in between 0.75 and 0.80 or invasive iFR values in between 0.86 and 0.89. These ranges fall within recognized “gray-zones”, in which determining the appropriateness of revascularization versus conservative medical treatment remains debatable [29]. A subgroup of 16 cases showed discordance between invasive FFR and iFR measurements, highlighting the difficulty of establishing an absolute reference standard, even with invasive techniques. In this discordant subgroup, CT-FFR_AI_ showed greater concordance with invasive FFR than with iFR, which may reflect the fact that CT-FFR_AI_ was trained using FFR as the reference standard. Given the clinical implications of false-negative findings and the diagnostic uncertainty in cases with discordant invasive measurements, outcome-based studies are warranted to further evaluate the clinical reliability of CT-FFR_AI_ [30].

As image quality may theoretically affect CT-FFR_AI_, we sought to evaluate the influence of patient-related and technical CCTA parameters on CT-FFR_AI_’s accuracy in predicting vessel-specific ischemia. The diagnostic performance of CT-FFR_AI_ was influenced by coronary calcifications, which may lead to underperformance in cases of heavily calcified coronary stenoses. However, in the intermediate stenosis range (26–75%), 53% of stenoses were correctly classified as true-negative, emphasizing the added value of CT-FFR_AI_. Since quantitative CCTA and ICA assessments poorly correlate with lesion-specific ischemia [31–33], CT-FFR_AI_ could play a significant role in ruling out ischemia and avoiding further invasive testing in this subgroup.

In a previous study, image quality was mainly influenced by the coronary diameter, while a higher heart rate and calcium score had a less negative impact [14]. The fact that other patient-related factors had no significant influence on the accuracy of CT-FFR_AI_ in our study may be related to a different selection process. The median vessel diameter was much higher (3.6 mm versus 2.8 mm), and the upper range of the heart rate during CCTA acquisition was lower (78 versus 110 bpm) in the present study, reducing the impact of motion effects on the image quality [14]. Novel techniques such as photon-counting CT, with higher spatial resolution, potentially reducing blooming artefacts, could reduce false-positive cases and improve the performance of CT-FFR_AI_ [34]. A promising first study reported excellent accuracy for predicting coronary artery disease from CT-FFR_AI_ in a setup of photon-counting imaging [26].

## Limitations

There are a few limitations to our study, first, its retrospective design and the inherent limitations of such studies. A prospective study with a similar sample size is required to confirm our results. Second, the CT-FFR_AI_ model relies on manually adjusted cMPR images, introducing susceptibility to the user, particularly in instances of centerline adjustment challenges, such as in examinations of poor quality or with heavily calcified lesions. An AI model incorporating automatic contouring and segmentation could mitigate operator dependency in FFR prediction. Third, our CT-FFR_AI_ model currently utilizes a dichotomous outcome with a threshold of 0.80, which overlooks clinical information pertaining to lesions in the gray zone. A continuous CT-FFR_AI_ outcome would be more clinically relevant. Fourth, CT-FFR_AI_ may not be valuable for assessing diffuse lesions. Fifth, the black-box nature of DLMs like CT-FFR_AI_ limits the interpretability; future developments could use heatmaps to highlight key features (e.g., lumen narrowing or calcification) driving predictions and thus offering more insight into their decision-making [9]. Lastly, our study did not investigate clinical outcomes, which is crucial information in the assessment of a new technique and should be evaluated in further studies.

## Conclusion

As compared to two validated pressure measurements during ICA, CT-FFR_AI_ has a high diagnostic accuracy in predicting vessel-specific coronary ischemia that was independent of the scanner type and supervisor’s level of experience. The extent of coronary calcifications significantly reduces the accuracy of CT-FFR_AI_, suggesting further developments in spatial resolution are needed to improve diagnostic precision, particularly in calcified vessels. The high sensitivity and NPV indicate that CT-FFR_AI_ may be particularly valuable in excluding ischemia-inducing lesions prior to invasive procedures.

## Supplementary information

Supplementary information

## Abbreviations

AI: Artificial intelligence
BMI: Body mass index
CAD-RADS: Coronary Artery Disease-Reporting and Data System
CCTA: Coronary computed tomography angiography
CFD: Computed fluid dynamics
CI: Confidence interval
cMPR: Curved multiplanar reconstruction
CT-FFR: FFR estimated from computational fluid dynamics simulations using coronary computed tomography angiography
CT-FFR_AI_: Coronary computed tomography angiography-based artificial intelligence deep-learning model for the prediction of invasive fractional flow reserve
DLM: Deep learning model
FFR: Fractional flow reserve
HU: Hounsfield units
ICA: Invasive coronary angiography
iFR: Instantaneous wave-Free Ratio
IQR: Interquartile range
NPV: Negative predictive value
OR: Odds ratio
PPV: Positive predictive value
SD: Standard deviation
SNR: Signal-to-noise ratio
