# Supplementary material for: Diagnostic performance of a coronary CT angiography-based deep learning model for the prediction of vessel-specific ischemia
Source: Eur Radiol. 2025 Oct 11;36(4):3054–65. doi: 10.1007/s00330-025-12048-4 (PMC13035628; doi:10.1007/s00330-025-12048-4)
Supplement: Supplementary file 1 — Supplementary information [file 330_2025_12048_MOESM1_ESM.pdf]

Supplementary Table A: - Diagnostic values of CT-FFR$_{AI}$ without supervision, compared to invasive FFR and iFR.

Diagnostic value % [95%CI]	FFR (n=224)	iFR (n=238)
Sensitivity	93(63/68) [84–98]	92(79/86) [84–97]
Specificity	47(73/156) [39–55]	45(69/153) [37–53]
PPV	43(63/146) [39–47]	48(79/163) [45–52]
NPV	94(73/78) [86–97]	91(69/76) [83–95]
Accuracy	61(136/224) [54–67]	62(148/238) [55–68]

The table shows the diagnostic values of CT-FFR$_{AI}$ without radiologists' supervision using invasive FFR and/or iFR as standard of reference for vessel-specific ischemia. When both invasive FFR and iFR values were obtained, they were analyzed independently.

CT-FFR$_{AI}$: coronary computed tomography angiography-based artificial intelligence deep-learning model for the prediction of invasive Fractional Flow Reserve, FFR: Fractional Flow Reserve, iFR: instantaneous wave-Free Ratio, cMPR: Curved Multiplanar reformatting, 95%CI: 95% confidence interval, PPV: positive predictive value, NPV: negative predictive value.
